# Collection of Annotated Acinetobacter Genome Sequences

**DOI:** 10.1128/mra.01094-22

**Published:** 2023-02-22

**Authors:** Macaulay Winter, Klaus Harms, Pål Johnsen, Michiel Vos

**Affiliations:** a European Centre for Environment and Human Health, University of Exeter Medical School, Environment and Sustainability Institute, Penryn Campus, United Kingdom; b Microbial Pharmacology and Population Biology Research Group, Department of Pharmacy, Faculty of Health Sciences, University of Tromsø-The Arctic University of Norway, Tromsø, Norway; Indiana University, Bloomington

## Abstract

The genus Acinetobacter contains environmental species as well as opportunistic pathogens of humans. Several species are competent for natural transformation, an important mechanism of horizontal gene transfer. Here, we present the genome sequences of 19 Acinetobacter strains used in past and upcoming studies of natural transformation.

## ANNOUNCEMENT

Acinetobacter species are ubiquitous and are found in both terrestrial and clinical environments (1). Many Acinetobacter strains are able to acquire new genetic information via natural transformation, and this ability is thought to be at least partly responsible for some clinically relevant Acinetobacter baumannii strains becoming multidrug resistant (2). Here, we sequenced the genomes of 19 Acinetobacter strains from a diverse set of environmental samples provided by David M. Young (Harvard University). These strains were previously used in a study investigating the efficiency of DNA uptake in different genomic locations by the model species for natural transformation, Acinetobacter baylyi (3).

All strains are culturable on LB agar or in LB broth at 37°C at 180 rpm. Genomic DNA of the strains was extracted from single colonies grown on agar using a DNeasy kit (Qiagen, Germany). Sequencing was carried out by the Centre for Genomic Research at the University of Liverpool. Short-read Illumina sequencing data (150-bp paired-end reads) were generated for each strain using the NEBNext Ultra II FS kit with an Illumina MiSeq system. The trimmed reads were quality controlled using FastQC (4), *de novo* assembled using Unicycler v0.4.8 (5, 6) with default parameters, and rotated to start at *dnaA*. Because contigs of <500 bp are usually artifacts of sequencing, they were omitted from downstream analyses. QUAST v5.2.0 was used to generate statistics for the assemblies (7). Genomic features were annotated using Prokka v1.13 (8). All strains had a linear genomic topology. Taxonomic classification of the strains was determined by topology and average nucleotide identity (ANI) values using GTDB-Tk v1.7.0 (9).

### Data availability.

The raw and annotated read data for the 19 strains were deposited in the European Nucleotide Archive (ENA) under BioProject accession number PRJEB55833. Accession numbers for raw reads and annotations are presented in Table 1.

**TABLE 1:** Accession numbers and sequencing data for raw reads and annotations

Strain name	Genome size (bp)	No. of contigs	Largest contig size (bp)	*N*_50_ (bp)	GC content (%)	GenBank assembly accession no. for fastANI reference	fastANI taxonomy (ANI [%])	No. of predicted genes (unique)	No. of reads	Avg read length (bp)	No. of sequenced bases	Whole-genome coverage (×)	Raw read accession no.	GenBank assembly accession no.
Acinetobacter sp. strain 01B0 Km^r^, isolate 2	4,012,069	99	390,125	119,727	38.93	GCF_009759685.1	Acinetobacter baumannii (97.75)	3,851	80,498,431	111.9	9,007,774,429	2,245.16937	ERR10167739	GCA_947331425
Acinetobacter sp. strain 26B2, isolate 3	4,124,650	18	1,229,448	1,063,596	38.48	GCF_000196795.1	Acinetobacter oleivorans (96.84)	3,817	74,338,568	114.25	8,493,181,394	2,059.127779	ERR10167740	GCA_947044995
Acinetobacter sp. strain 423D, isolate 3	4,125,066	17	1,229,448	1,063,963	38.48	GCF_000196795.1	Acinetobacter oleivorans (96.84)	3,816	61,075,879	121.7	7,432,934,474	1,801.894679	ERR10167741	GCA_947044985
Acinetobacter sp. strain 48A1, isolate 3	3,838,866	27	730,290	340,355	38.97	GCF_009759685.1	Acinetobacter baumannii (97.62)	3,616	50,129,031	116.55	5,842,538,563	1,521.943867	ERR10167742	GCA_947044945
Acinetobacter sp. strain 511B, isolate 5	4,124,706	18	1,229,448	1,063,596	38.48	GCF_000196795.1	Acinetobacter oleivorans (96.87)	3,817	61,442,508	114	7,004,445,912	1,698.168527	ERR10167743	GCA_947045055
Acinetobacter sp. strain 56A1, isolate 3	3,954,990	94	390,027	123,641	38.96	GCF_009759685.1	Acinetobacter baumannii (97.72)	3,789	51,944,110	114.25	5,934,614,568	1,500.538451	ERR10167744	GCA_947044975
Acinetobacter sp. strain 62A1, isolate 3	3,800,072	22	1,667,336	478,807	38.6	GCF_900520355.1	Acinetobacter calcoaceticus *C* (98.02)	3,526	55,955,595	114.1	6,384,533,390	1,680.108532	ERR10167745	GCA_947045035
Acinetobacter sp. strain 63A1, isolate 7	3,822,479	33	515,714	240,423	38.53	GCF_900520355.1	Acinetobacter calcoaceticus *C* (96.21)	3,525	63,908,045	102.75	6,566,551,624	1,717.87775	ERR10167746	GCA_947044905
Acinetobacter sp. strain 66A1, isolate 1	3,738,809	27	855,844	380,638	38.9	GCF_009759685.1	Acinetobacter baumannii (97.96)	3,440	45,304,263	118.3	5,359,494,313	1,433.476359	ERR10167747	GCA_947045065
Acinetobacter sp. strain 71A1, isolate 4	3,834,289	38	853,248	465,306	38.8	GCF_009759685.1	Acinetobacter baumannii (98.16)	3,608	51,498,217	114.25	5,883,671,292	1,534.488217	ERR10477801	GCA_947366405
Acinetobacter sp. strain 81A1, isolate 2	3,738,813	29	855,844	380,638	38.9	GCF_009759685.1	Acinetobacter baumannii (97.94)	3,438	43,162,178	119.05	5,138,457,291	1,374.355254	ERR10167749	GCA_947044935
Acinetobacter sp. strain 85A1, isolate 3	4,124,656	18	1,229,448	1,063,596	38.48	GCF_000196795.1	Acinetobacter oleivorans (96.86)	3,818	62,181,982	114.95	7,147,818,831	1,732.949083	ERR10167750	GCA_947045025
Acinetobacter sp. strain A06, isolate 7	4,124,674	18	1,229,448	1,063,596	38.48	GCF_000196795.1	Acinetobacter oleivorans (96.85)	3,814	44,354,854	116.8	5,180,646,947	1,256.013675	ERR10167751	GCA_947044965
Acinetobacter sp. strain A3-6, isolate 3	3,738,812	27	857,074	380,638	38.9	GCF_009759685.1	Acinetobacter baumannii (97.96)	3,440	40,888,945	118.3	4,837,162,194	1,293.769837	ERR10167752	GCA_947045075
Acinetobacter sp. strain AD512A, isolate 4	3,834,471	37	853,248	465,306	38.8	GCF_009759685.1	Acinetobacter baumannii (98.14)	3,609	49,769,835	120.6	6,002,242,101	1,565.337722	ERR10477802	GCA_947366465
Acinetobacter baumannii AZR3410, isolate 1	3,645,238	99	187,218	78,501	39.09	GCF_009759685.1	Acinetobacter baumannii (97.87)	3,488	57,196,478	116.3	6,651,950,391	1,824.832944	ERR10167754	GCA_947045015
Acinetobacter sp. strain AZR54, isolate 8	3,897,882	20	1,303,607	433,975	38.59	GCF_000368965.1	Acinetobacter calcoaceticus (99.98)	3,666	45,034,632	112.45	5,064,144,368	1,299.204124	ERR10167755	GCA_947044915
Acinetobacter calcoaceticus AZR583, isolate 9	3,787,947	38	445,412	238,481	38.56	GCF_000368965.1	Acinetobacter calcoaceticus (97.19)	3,566	54,949,893	112.75	6,195,600,436	1,635.609061	ERR10167756	GCA_947044925
Acinetobacter sp. strain P1-6, isolate 3	4,124,672	18	1,229,448	1,064,826	38.48	GCF_000196795.1	Acinetobacter oleivorans (96.88)	3,818	47,141,068	117.85	5,555,574,864	1,346.913128	ERR10167757	GCA_947044955
